# Insect-host control of obligate, intracellular symbiont density

**DOI:** 10.1098/rspb.2021.1993

**Published:** 2021-11-24

**Authors:** Mathilda Whittle, Antoine M. G. Barreaux, Michael B. Bonsall, Fleur Ponton, Sinead English

**Affiliations:** ^1^ School of Biological Sciences, University of Bristol, Bristol BS8 1TQ, UK; ^2^ Department of Zoology, University of Oxford, Oxford OX1 3PS, UK; ^3^ St Peter's College, Oxford, OX1 2DL; ^4^ Department of Biological Sciences, Macquarie University, Sydney, NSW, Australia

**Keywords:** symbiont density, regulation, host-control, cost-benefit, *Buchnera*, *Wigglesworthia*

## Abstract

Many insects rely on intracellular bacterial symbionts to supplement their specialized diets with micronutrients. Using data from diverse and well-studied insect systems, we propose three lines of evidence suggesting that hosts have tight control over the density of their obligate, intracellular bacterial partners. First, empirical studies have demonstrated that the within-host symbiont density varies depending on the nutritional and developmental requirements of the host. Second, symbiont genomes are highly reduced and have limited capacity for self-replication or transcriptional regulation. Third, several mechanisms exist for hosts to tolerate, regulate and remove symbionts including physical compartmentalization and autophagy. We then consider whether such regulation is adaptive, by discussing the relationship between symbiont density and host fitness. We discuss current limitations of empirical studies for exploring fitness effects in host–symbiont relationships, and emphasize the potential for using mathematical models to formalize evolutionary hypotheses and to generate testable predictions for future work.

## 1. Introduction

Mutually beneficial relationships between bacteria and animals are extremely common in nature. Beyond the diverse microbiota resident in the gut, positive associations with specific bacterial symbionts have been described for many insects [1]. Symbionts described as ‘obligate’ are required for host survival and reproduction and, likewise, typically cannot themselves live outside their host tissues. This contrasts with non-obligate (or ‘facultative’) symbionts, which, despite often providing benefits to the host, are not strictly required.

**Figure 1.**
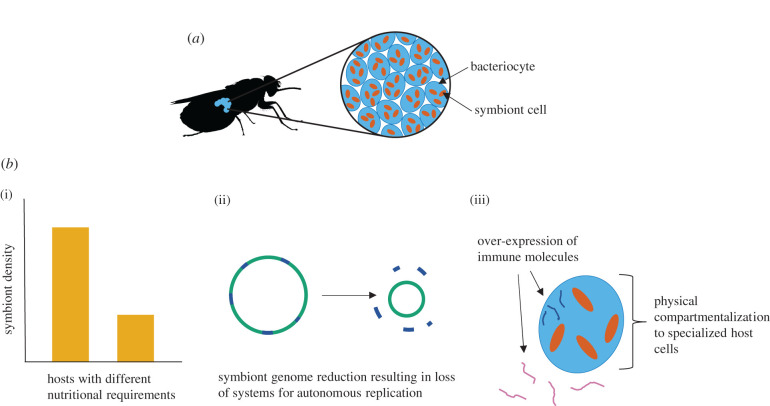
(*a*) Obligate, intracellular bacterial symbionts are located in the specialized host cells known as bacteriocytes. In tsetse, shown here as an example, the bacteriocytes are aggregated to form the bacteriome organ. (*b*) Three lines of evidence support the idea that hosts exert tight control over their symbiont density: (i) symbiont density often relates to the nutritional requirements of the host, for example, hosts may harbour different symbiont densities when provided with qualitatively different diets (e.g. [2]), between sexes (e.g. [3]) or throughout development [4]; (ii) symbionts have reduced genomes lacking systems for autonomous self-replication, for example, lacking genes for replication initiation (e.g. [5]); and (iii) several host mechanisms allow the symbiont population to persist while limiting symbiont proliferation. These include physical compartmentalization of the symbionts to bacteriocytes [6] and the production of immune molecules both within and outside the bacteriocytes (e.g. [7]). (Online version in colour.)

A characteristic of many obligate bacterial symbionts of insects is that the symbiont is located intracellularly within the host, in specialized and relatively large cells known as bacteriocytes (figure 1*a*) [8]. Most intracellular symbionts are acquired by host individuals via vertical transmission from parent to offspring [9]. In female hosts, symbionts are transmitted to developing offspring directly from the bacteriocytes (e.g. [10]), or from a secondary population of symbiont found within the reproductive tissues (e.g. [11,12]). This vertical transmission thus secures the symbiotic association for the next generation.

A universal function of obligate, intracellular symbionts is the provisioning of essential nutrients by the symbionts to their host, which are not available through host diet nor synthesized by the host. This has allowed the adaptation of many insects to feed on nutritionally limited diets consisting exclusively of one food type, for example, aphids on plant sap [13], termites on woody material [14] and tsetse on vertebrate blood [15]. Nutrient provisioning by symbionts supports development and reproduction (e.g. [16,17]), and the presence of symbionts during development can also benefit the host by priming the immune system (e.g. [18,19]). Symbiotic relationships are a potent driving force of adaptation and speciation, and the universal success and biodiversity of insects is owed, in part, to their bacterial partners [20,21].

As well as the benefits acquired by hosts of participating in mutualistic symbioses with bacteria, there are costs to hosts associated with providing the energy and nutrients to maintain a symbiont population (e.g. [22]). The net benefit to a host is therefore determined by the difference between the benefit and cost, with both benefits and costs potentially depending on the size of the symbiont population. As an example, the anti-viral protection provided by the symbiont *Wolbachia* to its hosts (e.g. *Drosophila* and *Aedes*) depends on the density of symbiont within the host [23,24]. Studies show that in infected hosts, the viral titre is negatively correlated with *Wolbachia* density [24], and that the anti-viral protection is not provided at low densities of symbiont [23]. Although studies have attempted to determine how costs and benefits scale with symbiont density (e.g. [25]), the functional forms of these relationships are not well understood. The net benefit of participating in a symbiosis is also dependent on the ecological context, which is subject to change. The ability of insects to regulate the density of their symbionts to levels which maximize the net benefit, tailored to specific ecological conditions, could therefore provide a fitness advantage.

In many insect systems, the density of obligate, intracellular bacterial symbionts appears to be actively regulated by the host in response to environmental and physiological factors. This review will examine the empirical support for this control (see figure 1*b* for a schematic), with particular focus on the potential costs and benefits in terms of host fitness associated with symbiont density. Further, this review will discuss the limitations in testing the adaptive nature of host regulation of symbiont density and present novel ideas about how the fitness effects underlying such regulation can be further investigated.

### (a) Dynamic symbiont density according to host requirements

Studies measuring symbiont density have found that it varies according to host requirements. Symbiont density can be quantified using flow cytometry to count the number of symbiont cells in the whole host or in the maternal bacteriocytes (e.g. [4]). More commonly, quantitative polymerase chain reaction (q-PCR) is used to quantify the ratio of symbiont genomes to host genomes (e.g. [26]) or host cell number (e.g. [3]). Although polyploidy is frequently observed among symbionts and ploidy can even change throughout host development [27,28], this is thought to be an appropriate measure of density owing to the likelihood of a strong correlation with the symbiont cell number and functional capacity [29].

#### (i) Diet

Owing to the nutritional exchange which forms the basis of intracellular symbiosis in insects, there is a tight interplay between host diet and symbionts. Tsetse (family Glossinidae) are host to one obligate symbiont of the genus *Wigglesworthia*. This symbiont provisions the host fly with thiamine [30], vitamin B_6_ [31], folate [32] and other nutrients complementing the exclusive vertebrate blood diet of tsetse, known to have low concentrations of these B vitamins [33]. Similarly, the majority of aphids harbour one obligate symbiont, *Buchnera*. This symbiont provides their host with essential amino acids (EAAs) and B vitamins available at insufficient concentrations in their phloem sap diet [34].

**Figure 2.**
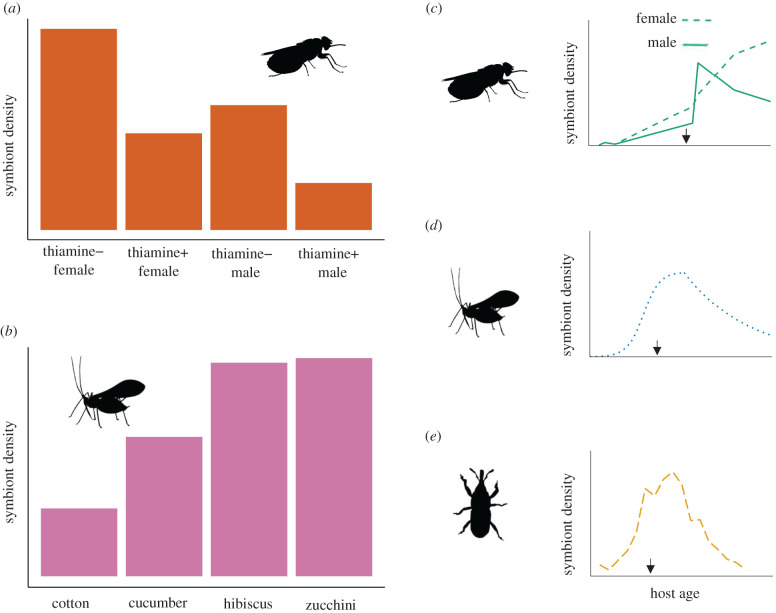
(*a*) Effect of dietary supplementation with a thiamine (vitamin B_1_) derivative on symbiont density in tsetse [2]; (*b*) effect of host plant on symbiont density in cotton-melon aphids [35]; (*c*–*e*) patterns of symbiont density change observed throughout the lifetime of various insect hosts; (*c*) tsetse [3]; (*d*) pea aphid [4]; (*e*) cereal weevil [36]. Arrows indicate the onset of maturity. All plots (*a*–*e*) are reproductions based on data extracted from the figures of cited papers. (Online version in colour.)

Studies on both tsetse and aphids have measured the effect of experimental manipulation of host nutrition on symbiont density. Nutritional supplementation of the tsetse diet with a thiamine derivative results in a decreased density of *Wigglesworthia* in both male and female *Glossina morsitans* (figure 2*a*) [2]. Receiving an enriched diet may result in the host having a reduced requirement for the symbiont-provisioned nutrient and, therefore, a reduced requirement for *Wigglesworthia* itself [2]. By contrast, *Acyrthosiphon pisum* aphids reared on a nitrogen-rich diet had a higher density of *Buchnera* than those fed on a nitrogen-poor diet [37]. The aphids reared on nitrogen-rich diets grew at a much greater rate than those on nitrogen-poor diets. This may have resulted in a higher demand for the *Buchnera*-provisioned nutrients to support this rapid growth and, therefore, a requirement for greater densities of *Buchnera* [37].

The results of these dietary supplementation experiments show an interesting contrast. On the one hand, supplementation of the host diet with symbiont-derived nutrients (e.g. tsetse with thiamine) has a negative effect on symbiont density. On the other, increasing the quality of the host diet without supplementing with symbiont-derived nutrients (e.g. aphids with nitrogenous compounds but not EAAs) has a positive effect on symbiont density. The former could be a host-mediated mechanism of reducing the costs of maintaining a symbiont population superfluous to requirements, and the latter a way of maximizing the nutritional output, and therefore the benefit, from the symbiont population.

Diet has also been shown to influence symbiont density in a more natural setting. The density of *Buchnera* in *Aphis gossypii* varied among aphid groups reared on different plants (figure 2*b*). When transplanted onto different host plants, the density of the symbiont adjusted to new levels within the aphids [35]. Changes in the *Buchnera* density could have been owing to chemicals in the plants promoting or suppressing *Buchnera* growth directly. Alternatively, the readjustment of symbiont density upon introduction to new host plants could be mediated by the aphid hosts in response to the individual nutrient profiles of the different plants [35]. The latter explanation is consistent with the aforementioned experimental studies on the effect of diet on symbiont populations, whereby insect hosts actively adjust the density of symbionts in response to nutrient availability and their dietary requirements [2,37]. Importantly, aphid survival generally decreased upon introduction to new host plants, which then improved and stabilized after several generations [35], indicating a relationship between *Buchnera* density and host fitness.

Studies comparing dietary EAA requirements of different *Ac. pisum* clones support the suggestion that hosts maintain symbiont levels according to their individual nutritional needs. Dietary EAA requirements were identified by comparing the mass of the aphids fed on a full diet with those fed on a diet lacking EAAs [38]. The results showed that the different clones have different requirements for dietary EAAs, and a subsequent study determined that these requirements were moderately and positively correlated with the density of *Buchnera* found in these hosts [39].

The results of these studies indicate that the dietary requirements of insects have a significant effect on the density of their symbionts, and hosts regulate symbiont density in accordance with the level which provides the most benefit. The interaction between symbiont density and diet is well explored in herbivorous insects such as aphids; however, there are limited studies using other systems (but see [2]). There is an opportunity to investigate these effects in hosts with diverse diets, such as insects which feed on blood and wood.

#### (ii) Sex and development

In the case of vertically transmitted obligate symbionts, offspring tend to start life with a low symbiont density as the symbiont titre transmitted to offspring is generally small in proportion to the amount mothers contain (e.g. [40,41]). In *G. morsitans*, *Wigglesworthia* density remains at this relatively low level during the larval and pupal stages and increases rapidly at the start of adulthood. In newly emerged (i.e. teneral) males, the density of *Wigglesworthia* reaches a maximum and then decreases during adulthood (figure 2*c*) [3,26]. Fully mature adult males may no longer have a high demand for symbiont-derived nutrients and may therefore reduce the density of their symbiont in order to minimize the cost of supporting the symbiont population.

The density of *Wigglesworthia* in adult female tsetse is greater than that of males [2] and increases continuously from the start of adulthood (figure 2*c*) [3,26]. This sex difference is reflected in the relative sizes of the bacteriome (the symbiont-housing organ made up of bacteriocytes), which decreases in males during adulthood, but not in females [42]. Female tsetse invest a large amount of energy and nutrients into reproduction, as they produce relatively large offspring continuously across their adult life [43,44]. A large and constant supply of symbiont-derived nutrients may therefore be required to support the huge energetic demands of reproduction [3]. However, the continuous increase in symbiont density of adult tsetse females raises questions. If, during adulthood, a particular symbiont density is optimal for survival and reproduction, why does the density not increase more rapidly at the start of adulthood (as is observed in males, figure 2*c*), and then plateau at the optimal level? While no studies have addressed this, we could hypothesize that the absence of a plateau is a case of tsetse minimizing the costs associated with regulating the symbiont population. As tsetse females get older, and some physiological functions potentially deteriorate [45], the cost of limiting the proliferation of *Wigglesworthia* might become greater than the cost of supporting a large population, and so the symbiont population is allowed to continue to grow with minimal regulation. An alternative possibility is that the optimal *Wigglesworthia* density is particularly high and is not reached within the age range of female hosts tested in this study [3]. The slow proliferation of *Wigglesworthia* may then be the result of a compromise between what the host can afford to invest in increasing the symbiont density and other energetic demands. Finally, the optimal symbiont density could be dynamic, and the observed pattern of symbiont density increase is a reflection of the continuously increasing requirement for symbiont-derived nutrients.

Examples of sustained symbiont proliferation during adulthood are known in other insects. The Asian citrus psyllid, *Diaphorina citri*, harbours two obligate symbionts in its bacteriocytes, *Carsonella* and *Proftella*, which both demonstrate a pattern of density increase similar to that observed in female tsetse [46]. The underlying mechanisms determining the continuous increase in symbiont density during adulthood of these hosts merit further investigation.

Female aphids show patterns of symbiont density change similar to that of male tsetse. *Buchnera* cells in the maternal bacteriocytes of parthenogenic *Ac. pisum* show a rapid proliferation in number during nymphal development, reaching a stationary phase in early adulthood and then decreasing during later ages (figure 2*d*) [4]. Changes to the bacteriocytes throughout aphid development mirror the dynamics of the symbiont population [4]; the bacteriocytes increase in size and number, reaching a maximum at the beginning of adulthood and thereafter degrading and becoming fewer in number. The mode of reproduction in these aphids may explain the dissimilarity with female tsetse; the parthenogenic offspring are laid in a single period of about 9 days, starting at the beginning of adulthood. The beginning of the laying period marks a point in the lifetime of the mother where there is no longer a high metabolic demand to support embryogenesis, and the subsequent decrease in symbiont density may occur in order to minimize costs of maintaining a large symbiont population [4]. Whether there is a delay to the decrease in symbiont density upon reaching the laying period, or if this occurs immediately, is unclear.

The trend of symbiont proliferation and decline has also been observed in carpenter ants (*Camponotus* spp.), which harbour the symbiont *Blochmannia* [47]. An increase in *Blochmannia* density during early life, followed by a decrease in late adulthood, has been observed in both males and workers of *Camponotus floridanus* [48–50]. In addition, microscopic techniques revealed that young adult individuals show bacteriocytes full of symbionts; however, old individuals show few, if any, bacteriocytes harbouring very few bacterial cells [48]. Perhaps surprisingly, the bacteriocyte-associated symbiont population in *C. floridanus* queens also undergoes severe depletion during maturity [51], indicating that a symbiont population is not necessarily required to meet the nutritional demands of reproduction. A separate population of the symbiont is harboured by females in the reproductive tissues, the levels of which remain constant throughout ageing in reproductive workers [51]. Thus, the transmission of *Blochmannia* to progeny can be ensured despite the depletion from maternal bacteriocytes. The extreme decline in numbers of *Blochmannia* in carpenter ants indicates that the role of this symbiont is of most importance during the early stages of development [48]. Accordingly, the removal of *Blochmannia* from non-reproducing adults using antibiotics does not result in any noticeable negative effects on feeding behaviour or host appearance [51].

Cereal weevils present yet another example of symbiont growth and decline during host development. In *Sitophilus oryzae*, the total population size of the obligate symbiont *Sodalis* increases dramatically between the final moult and early adulthood, followed by a decrease and ultimately, removal of the symbiont from the bacteriocytes (figure 2*e*) [36]. The population of *Sodalis* residing in the ovaries of females is not observed to deplete during adulthood, ensuring the transmission of this symbiont to progeny. As with carpenter ants, the near-complete removal of the symbiont from the bacteriocytes of cereal weevils may reflect the specific function of this symbiont in its respective host; rearing weevils devoid of symbionts (i.e. aposymbiotic) results in hosts with paler and softer cuticles, indicating a role of *Sodalis* in cuticle development. After maturation of the cuticle, it could be that *Sodalis* becomes redundant and is removed from the bacteriocytes in order to minimize the cost of maintaining the symbiosis for the host [36]. Likewise, the removal of *Blochmannia* in carpenter ants may serve to minimize the cost of maintaining a symbiont population which is no longer providing a benefit to the host. By contrast, *Buchnera* and *Wigglesworthia* presumably have lifelong roles in the biology of aphids and tsetse, hence the presence of these symbionts throughout the lifespan of their hosts.

From the insect systems reviewed herein, three distinct patterns of intracellular symbiont density dynamics emerge: sustained symbiont proliferation throughout host development (e.g. female tsetse and psyllids); symbiont density increase in early adulthood, followed by a reduction in symbiont density (e.g. male tsetse and aphids); and finally, symbiont density increase in early adulthood, followed by symbiont removal or near-removal (e.g. carpenter ants and cereal weevils). Explanations for these trends have been proposed by considering the benefits and costs of the symbiosis to the host, as well as how the nutritional requirements of the hosts may change throughout development and with respect to reproduction.

### (b) Reduced functionality of symbiont genomes

The observation that symbiont density is frequently correlated with the developmental and nutritional needs of hosts reinforces the idea that many insects regulate the density of their symbionts. Genome sequencing has been used to determine the ability, or lack thereof, of density control by the obligate intracellular symbionts themselves. Analysis of the *Buchnera aphidicola* genome has shown that although it retains most of the genes encoding enzymes for EAA biosynthesis, it lacks nearly all ancestral regulatory mechanisms, including genetic systems for controlling gene expression in response to environmental changes [52,53]. As it would be disadvantageous to the host for symbionts to produce excess nutrients, or use precursor nutrients which are limiting, it could be presumed that the host exerts regulation of certain biosynthetic pathways. Sequencing of the *Blochmannia floridanus* genome revealed that this symbiont does not possess any known mechanisms of replication initiation [5,54], and likewise, *Wigglesworthia glossinidia* lacks the gene encoding DNA replication initiation protein *DNAa* [55]. This loss of autonomous DNA replication mechanisms within symbionts suggests that these are host-mediated processes, which would provide the host with strict control of symbiont replication, population-level growth and, hence, density.

Obligate, intracellular symbionts share long coevolutionary histories of up to greater than 260 million years with their respective insect hosts [56], which has resulted in the characteristically reduced genomes of these symbionts (figure 1*b*) [57]. Small populations of clonal symbionts are transmitted between hosts via the maternal line, which results in relaxed selection on the symbionts and increases the influence of genetic drift [58]. As a result, the rate of fixation of deleterious mutations causing gene loss and inactivation is elevated [59]. As the symbiosis contributes significantly to host fitness, there is selection on hosts to compensate for this loss of symbiont functionality via systems to support symbiont growth and function. As hosts evolve to compensate for symbiont deficiencies, genome decay is further facilitated and explains the tiny genomes with limited functionality observed in ancient symbioses [60]. It is possible then that the high level of control that hosts appear to exert over their symbiotic partners is not selected for directly, but is a by-product of the selection on hosts to compensate for deteriorating symbiont genomes.

This raises the additional question: are the benefits to the host of the symbiotic partnership greater when the host must compensate for the symbiont gene loss, or when the host is involved in a true mutualism with autonomous bacteria? Ankrah *et al.* [61] used metabolic modelling of the nutrient exchange between various hemipterans and their symbionts to indicate that that the metabolic costs of maintaining a symbiont population is lower for symbionts with smaller genomes. This was attributed to the higher productivity of symbionts with smaller genomes, which appear to have simpler metabolic networks and produce EAAs from host-provisioned precursors with greater efficiency. Thus, in addition to the relaxed selection on symbionts, increased host control of symbiont density might evolve owing to selection on hosts to minimize metabolic costs [61].

Here, we consider only the benefits and costs to individual hosts of participating in such symbioses. On an evolutionary timescale, the limitation to niche environments and the increasing dependency on their obligate symbionts could be disadvantageous to insect species, for example, by potentially increasing the risk of extinction [60].

### (c) Host-mediated mechanisms of symbiont control

The host cells and structures which house obligate symbionts have evolved independently multiple times in invertebrates and display diverse morphology among taxa [1]. In many hosts, the bacteriocytes are aggregated to form the bacteriome, which can vary in location and structure, even between phylogenetically closely related taxa [62,63]. In other cases, bacteriocytes can be found individually, distributed throughout the fat body, as is seen in cockroaches and termites [64,65]. Despite this diversity, the compartmentalization of symbionts to specialized cells appears to serve three main functions relating to the control of symbiont density (figure 1*b*): (i) to provide a physical limit to the proliferation of symbionts, (ii) to protect symbionts from the host immune system, thus allowing them to persist, and (iii) to prevent symbionts from successfully infecting other host tissues, via the expression of immune molecules. Chomicki *et al.* [6] provide an in-depth discussion of such compartmentalization as an adaptive mechanism for host-mediated control of mutualisms.

Immune functions can aid the compartmentalization of symbionts to the bacteriocytes. Studies on gene expression patterns in the bacteriome of the cereal weevil *Sitophilus zeamais* revealed that the anti-microbial peptide (AMP)-encoding gene, *coleoptericin A* (*ColA*), was over-expressed in the bacteriome [66]. Silencing *ColA* in *Sitophilus* spp. resulted in the symbiont Sitophilus Primary Endosymbiont (SPE) escaping from the bacteriocytes, indicating that this molecule acts to prevent tissue invasion and colonization by this symbiont [7]. The anti-microbial activity of *ColA* was tested on *Escherichia coli* and was shown to inhibit cell division, resulting in bacterial gigantism [7]. Furthermore, injection of SPE into the haemolymph of *S. zeamais* resulted in the upregulation of genes encoding AMPs in the bacteriome [66]. Similarly, injection of *Blochmannia* into the haemocoel of *C. floridanus* triggered an immune response comparable to infection with other bacteria [67]. These results indicate that—outside of the bacteriocytes—these symbionts are recognized by the hosts as intruders, and that bacteriocytes serve to shield the symbiont from inducing an immune response within the host (figure 1*b*).

Peptidoglycan recognition proteins (PGRPs) are a family of proteins which modulate the immune response upon recognition of bacterial peptidoglycans [68]. In tsetse, a positive correlation between PGRP-lb expression levels and *Wigglesworthia* density has been observed, and the silencing of PGRP-lb results in a significant decrease in *Wigglesworthia* density in comparison to controls [69]. The catalytic activity of PGRP-lb is assumed to suppress the activation of immune responses to *Wigglesworthia* by breaking down immune-triggering peptidoglycan [70]. The preferential expression of PGRP-lb in the bacteriocytes thus enables the symbiont to persist within these cells (figure 1*b*) [71].

Autophagy has an important function in the immune response of insects, whereby the engulfment and digestion of microbes by enzyme-containing lysosomes removes these unwanted organisms from host cells. In aphids, degradation of *Buchnera* is observed to occur via activation of the lysosomal system in adulthood [72]. Nishikori *et al.* [73] reported that around the time of the final moult, the density of *Buchnera* and the total volume of bacteriocytes decreased in the winged *Ac. pisum* morph, corresponding to a period when the flight muscles develop rapidly. Recycling some *Buchnera* cells and bacteriocytes via digestion with the lysosomal system could support the temporary increase in metabolic activity. Such recycling would also reduce the cost of maintaining the symbiont population during a time of high nutritional demand. Similarly, after developing their cuticle, *Sitophilus* spp. weevils remove redundant symbionts with coordinated apoptosis and autophagy [36]. These mechanisms of symbiont removal can thus serve to recycle symbiont materials, thereby returning some of the investment back to the host.

### (d) Fitness consequences of symbiont density in hosts: empirical constraints and theoretical insights

The results of the experimental studies reviewed herein support the idea that insects have tight control of their symbiont densities, and that these densities are regulated by the hosts in response to their developmental and nutritional requirements. This leads to the central question of whether such symbiont regulation maximizes the net fitness benefit for hosts participating in a symbiosis. In other words, is the active regulation of symbiont density adaptive for hosts? Measuring host fitness in the absence of symbionts is relatively straightforward, as aposymbiotic hosts can be reared by treating mothers with antibiotics. The results of these treatments clearly demonstrate that reproduction, development and immunity are severely compromised in the absence of symbionts [22,74–77]. More challenging is manipulating symbiont density and measuring effects on host fitness. Only one study, to our knowledge, has attempted to assess the correlation between host fitness and non-zero symbiont density, by determining that different clone lines harboured different densities of *Buchnera* in fourth-instar aphids [29]. For each clone line, measures of host fitness, in terms of fecundity, developmental time and time to first reproduction, were correlated with the previously found symbiont densities. It was shown that higher symbiont density was moderately and negatively correlated with host performance, potentially a result of the cost of supporting larger symbiont populations [29].

To our knowledge, no other experimental studies of the symbiont density dependence of host fitness in an obligate intracellular insect symbiosis have been undertaken. There is a limitation in directly testing the fitness effect of non-zero symbiont densities on insect hosts, as current methods of quantifying symbiont density within individuals rely on killing the hosts. Consequently, the measurement of fitness traits of the same individuals is prevented.

In lieu of feasible experimental procedures, theoretical approaches allow us to explore many questions in biology [78,79]. In particular, exploration of the nature of nutritional symbioses in insects has benefited from mathematical modelling. Metabolic models built using genomic data have produced quantitative predictions of the costs and benefits (in terms of nutrient exchange) for hemipteran hosts and intracellular symbionts involved in a nutritional symbiosis [61,80]. Metabolic modelling of nutrient production by *Buchnera* indicates that the rate of production of EAAs by the symbiont is largely controlled by the supply of precursors from the aphid host [81]. Using functional transport data, Price *et al.* [82] proposed a model for substrate feedback inhibition of EAA production by *Buchnera*, whereby the accumulation of arginine in the haemolymph competitively inhibits the transport of precursor glutamine into the bacteriocytes, thus regulating the biosynthesis of this EAA. These results have expanded our understanding of the mechanisms by which hosts control the activity of their symbionts, according to their own requirements.

To date, we are not aware of any theoretical approaches that have investigated the density dependence of host fitness in insect-obligate symbiont systems, although this has been applied to other invertebrate symbioses. Cunning *et al.* [83] used cost-benefit modelling to determine the optimal density (i.e. that which maximized the net benefit in terms of nutrient production) of symbiont in an algae-coral symbiosis. The results support the idea that coral regulates their algal symbionts in response to environmental conditions. Dean *et al.* [84] modelled the effect of symbiont density on host fitness in a ciliate-*Chlorella* photosynthetic endosymbiosis, accounting for nutrient exchange and host control of symbiont density. The model predicts that the optimal symbiont density is dependent on the intensity of light.

The putative level of control which insects have over the density of their obligate intracellular symbiotic partners opens up opportunities for using optimality modelling to help understand host regulation of symbiont populations. For a given host symbiont system, modelling the context and density dependence of costs and benefits, in terms of host fitness, would allow predictions to be made on the symbiont density which maximizes the net benefit for hosts. These predictions could be made under different conditions, for example, varying temperature or nutrient availability. Comparison of model predictions with observed symbiont densities could provide a basis to assess whether hosts optimize symbiont density. Additionally, dynamic optimization modelling could provide insight into the potential strategies hosts should take to regulate symbiont density for the maximum fitness benefit. Such modelling would generate testable predictions about the optimal density of symbionts and patterns of host regulation, which could be compared against empirical evidence. The intimacy of the relationship between insects and their obligate intracellular symbionts renders many questions about these symbioses difficult to approach empirically, and applying theoretical methods could help open new avenues for research and inspire interesting new questions.

## 2. Conclusion

Traditionally viewed as mutualistic, several aspects of the relationship between insects and their obligate intracellular bacteria are believed to be largely under the control of the host, including the density of symbiont populations residing within host individuals. Empirical studies have provided considerable insight into the mechanisms and dynamics of symbiont regulation in a variety of insect systems, and how variation in symbiont density often corresponds to the nutritional and developmental requirements of hosts. However, there are gaps in our knowledge of how the density of symbionts affects host fitness. Owing to the intimacy of these symbioses, current experimental procedures limit our ability to investigate the context and symbiont density dependency of host fitness empirically. Theoretical approaches offer a powerful tool for investigating aspects of these symbioses, for example, the fitness effects of symbiont density under different contexts and the adaptive strategies taken by hosts in regulating symbiont populations.

## Data accessibility

This article has no additional data.
